# Risks of cardio-vascular diseases among highly active antiretroviral therapy (HAART) treated HIV seropositive volunteers at a treatment centre in Lagos, Nigeria

**DOI:** 10.11604/pamj.2021.38.206.26791

**Published:** 2021-02-23

**Authors:** Oloruntoba Ayodele Ekun, Emmanuel Olusesan Fasela, David Ayoola Oladele, Gideon Odemakpore Liboro, Toyosi Yekeen Raheem

**Affiliations:** 1 Department of Medical Laboratory Science, College of Medicine, University of Lagos, Idi-araba, Lagos State, Nigeria,; 2 Clinical Diagnostic Laboratory, Nigerian Institute of Medical Research, Yaba, Lagos State, Nigeria,; 3 Clinical Science Department, Nigerian Institute of Medical Research, Yaba, Lagos State, Nigeria,; 4 Center for Human Virology and Genomics, Nigerian Institute of Medical Research, Yaba, Lagos State, Nigeria

**Keywords:** Cardiovascular disease, highly active antiretroviral therapy, dyslipidemia, cardiac risk ratio, QRISK3

## Abstract

**Introduction:**

highly active antiretroviral therapy (HAART) has led to a decline in HIV-induced morbidity and mortality in recent years. However, it has been opined that this has led to elevated risks of cardiovascular diseases (CVDs). This study assessed the risks of CVDs among HAART experienced individuals living with HIV.

**Methods:**

a cross sectional study involving 196 adults consisting of 118 HAART experienced and 78 HAART naïve was conducted. Anthropometric and blood pressure measurements were recorded for all participants. Blood samples obtained from the volunteers were used to determine glucose, creatinine, HIV viral load, CD4 count and lipid profile using standard methods. Lipid ratios, atherogenic indices and QRISK3 risk score were calculated.

**Results:**

the median CD4 lymphocyte, mean body mass index (BMI) and HDL-c in HAART experienced participants were higher (P<0.05) than HAART naive individuals. The QRISK3 risk score and creatinine were higher (p<0.05) among HAART experienced group. In HAART experienced group, the risk of hypertension, increased low-density lipoprotein (LDL-c), atherogenic index of plasma (AIP) and QRISK3 were 3.7, 2.0, 2.38 and 3.85 times (p<0.05) higher respectively than in HAART naive. Atherogenic coefficient (AC) increase was more prevalent among male (p<0.05) participants. Risk of chronic renal disease (eGFR), hypertension and CVD (as measured by QRISK3) was higher (p<0.05) among the female and older participants respectively.

**Conclusion:**

the risk of CVDs and renal disease appeared to be higher among HAART experienced volunteers and older (>45 years) volunteers. The risk of renal disease appeared higher in females.

## Introduction

Gradual deposition of plaque in the coronary artery in the form of foam cells results in cardiovascular disease, sequel to the formation of fatty streaks, leading to the development of atherosclerosis and finally to sudden cardiac death [1]. In human immunodeficiency virus (HIV) infected individuals, injuries to endothelial cells result in atherosclerosis which impacts an increased risk of cardiovascular diseases (CVD) and derangements in blood coagulation [2]. The risk of cardiovascular diseases increases with the presence of components of metabolic syndrome, malnutrition and smoking [3].

Dyslipidaemia is characterized by abnormal plasma concentration of lipid components. There is a strong association between the incidence of CVD and abnormal levels of LDLC and high-density lipoprotein (HDLC); the former being abnormally high while the latter is abnormally low. The LDLC/HDLC ratio is often calculated to estimate the cardiovascular risk [4]. On the other hand, it has been reported that a high level of triglyceride (TG) and increased LDLC particles were associated with increased cardiovascular risk. Thus, atherogenic dyslipidaemia (AD), which is defined as high LDLC/HDLC ratio and hyper-triglyceridemia (TG), is associated with high cardiovascular risk.

Currently, emerging lipid ratios such as Castelli´s risk index I and II (CRI-I and CRI-II), atherogenic risk of plasma (AIP) and atherogenic coefficient (AC) have been used to assess or predict the future risk of cardiovascular diseases [5]. There are limited studies on the utility of these ratios among HIV individuals on HAART in Nigeria. This study therefore focuses on assessment of lipid and various lipid ratios (Castelli´s risk index (I and II), atherogenic risk of plasma (AIP), atherogenic coefficient (AC)) as predictors of cardiovascular disease in HIV patients in Nigeria. Recent data have shown that CRI-I and CRI-II (estimated as TC/HDLc and LDLc/HDLc ratios respectively) are more accurate predictors of cardiovascular risk than traditional lipid parameters. CRI-I and CRI-II have been reported to be involved in predicting the risk of CAD [5]. Changes in these ratios have been reported to be better predictors of successful coronary heart disease (CHD) risk reduction when compared with changes in absolute levels of lipids or lipoproteins [6]. This remains to be comprehensively examined among HIV seropositive individuals on HAART in Nigeria.

In addition to this, the atherogenic risk of plasma (AIP) (defined as log10 (TG/HDLc)) was initially proposed by Dobiasova and Frohlich [7], as a marker of plasma atherogenicity. Previous studies showed that AIP is a useful diagnostic tool when information from other atherogenic risk factors are inconclusive [7,8]. Thus, AIP seems to be highly important in predicting future CVD episodes and for effective therapeutic monitoring [7]. This however remains to be thoroughly studied among HAART experienced and HAART naïve Nigerians. Furthermore, atherogenic coefficient (AC), (estimated as {(non-HDLc)/HDLC} or {(TC-HDLc)/HDLC}) has been described as a possible marker of cardiovascular diseases. Hermans *et al*. [9] reported that non-HDLc mimics the role of Apo-B in determining atherogenicity level as well as lipoprotein burden and consequently termed a valid surrogate of Apo-B apolipoprotein. This study, therefore, assessed lipids and lipid ratios as cardiac risk factors among HIV seropositive HAART-experienced and HAART naïve Nigerians.

## Methods

**Study design and study site:** this was a cross-sectional and observational study conducted at the Nigerian Institute of Medical Research (NIMR), Lagos, Nigeria.

### Study participants

**Sampling techniques and study procedure:** study participants were selected through a systematic random sampling technique using daily attendance as the sampling frame. Participants were recruited on each clinic day if they meet the inclusion criteria and signed a written informed consent to participate in the study. This study complied with the Helsinki Declaration.

### Data collection

**Numerical risk calculations:** estimated risk of CVD, was calculated using QRISK3 risk score (QRISK3)-a computerised mathematical model calculator. The QRISK®3 2018 calculator was developed for use in the UK, but is being used internationally. The QRISK®3 algorithm, calculates an individual´s risk of developing a heart attack or stroke over the next 10 years by answering simple questions. The results were stratified in levels in which a risk from 0-10% was considered as low risk, 11%-20% intermediate risk, and >20% high risk. The QRISK®3 - 2018 has an advantage that it was adjusted for ethnicity and so can be used for the purpose of risk of cardiovascular disease (CVD) among black Africans.

### Selection criteria

**Inclusion criteria:** consenting patients living with human immunodeficiency virus (HIV) attending NIMR PEPFAR clinic at the time of study aged 25 to 84 years were included in this study. This selection was based on the mathematical model used to calculate the CVD risk (QRISK®3 2018 algorithm). Both patients who were HAART experienced and HAART naïve were included in the study. The HAART experienced were considered as volunteers who had been on HAART treatment for at least 3 months and above while the HAART naïve volunteers were those who were just diagnosed of human immunodeficiency virus and were about been enrolled on HAART as at the time of their enrollment into this study. Only those who signed a written informed consent to participate in the study were recruited into the study.

**Exclusion criteria:** all pregnant and lactating women living with human immunodeficiency virus were excluded from this study. All known hypertensive, diabetes mellitus patients living with human immunodeficiency virus at the time of enrolment for this study were excluded. Volunteers with history of CVDs in a first degree relative at the point of enrolment were also excluded from this study. HIV exposed individuals under the age of 25 and non-consenting participants were all excluded from this study.

**Data analysis:** data generated from this study were analysed using a computer program statistical package for the social science (IBM SPSS version 20.0 statistics). Continuous variables from the subgroups were compared using mean and standard deviation (for normally distributed data) or median and interquartile range (IQR) (for skewed data), while categorical variables were recorded as frequencies and percentages. Chi-square test was used to test for the difference between categorical variables, whereas the difference between continuous variables was analysed using independent t-test or mann-whitney U test as appropriate. Pearson´s correlation coefficient was used to assess correlations between lipid ratios and clinical parameters.

**Ethical clearance:** ethical clearance to conduct the study was obtained from NIMR Ethics Review Board. The study was in accordance with Helsinki Declaration. Detailed information about the study was given to the patients and written informed consent obtained from the individuals who accepted to participate in the study.

**Anthropometric measurement:** height and weight were measured using standard meter tape and standiometer respectively. The BMI was calculated. Participants´ blood pressure was also measured and recorded.

**Laboratory procedures:** venous blood samples were collected for viral load, CD4, T-lymphocyte, glucose, creatinine and lipid profile. Castelli´s risk index-I (CRI-I) also known as cardiac risk ratio (CRR), Castelli´s risk index-II (CRI-II), atherogenic index of plasma (AIP), atherogenic coefficient (AC) [5,7] and eGFR were all calculated. Plasma samples were analyzed for blood glucose, creatinine and lipid profile (total cholesterol, triglyceride, HDL-cholesterol and LDL cholesterol) using Cobas C311 (Roche Diagnostics, Germany) according to instrument manuals (Roche C311 analyzer´s operator´s manual), whole blood samples were analysed for CD4 T-lymphocyte cells using Cyflow machine (Partec Cyflow counter, Germany) and plasma sample for HIV (RNA) viral load using Cobas Ampliprep/Taqman.

## Results

A total number of 196 volunteers consisting of 78 HAART naïve and 118 HAART experienced groups participated in this study. The HAART naïve group consists of 30 (38.5%) males and 48 (61.5%) females while the HAART experienced group was made up of 41 (34.7%) males and 77(65.3%) females respectively (Table 1). The mean age (year) of HAART naïve group was 36.15 ± 9.69 while HAART experienced group was 46.14 ± 9.0. The mean weight (Kg) and body mass index (BMI) (Kg/m^2^) of the HAART naïve group and the HAART experienced group were: 67.06 ± 15.37Kg, 75.75 ± 16.76Kg and 25.02 ± 5.15Kg/m^2^, 27.97 ± 6.44Kg/m^2^ respectively. The mean systolic and diastolic blood pressure (mmHg) were 117.18 ± 16.70; 128.75 ± 18.88 and 74.83 ± 11.85; 82.69 ± 11.56 respectively.

**Table 1:** comparative analysis of the demographic and blood pressure measurement among the studied participants

Parameter	HAART naïve group (n=78)	HAART experienced group (n=118)	p-value
**Characteristics**			
Mean age (years)	36.15 ± 9.69	46.14 ± 9.00	<0.001*
Mean height (meter)	166.58 ± 9.21	162.16 ± 22.78	0.023*
Mean weight (kg)	67.06 ± 15.37	75.75 ± 16.76	<0.001*
Mean BMI (kg/m2)	25.02 ± 5.15	27.97 ±6.44	0.032*
Mean SBP (mmHg)	117.18 ± 16.70	128.75 ± 18.88	0.020*
Mean DBP (mmHg)	74.83 ± 11.85	82.69 ± 11.56	<0.001*
**Sex**			
Male (%)	30 (38.5)	41 (34.7)	0.352
Female (%)	48 (61.5)	77 (65.3)	
Participants in %	78 (39.8%)	118 (60.2%)	

P-value <0.05 is considered statistically significant

A comparative study of the lipid and biochemical profiles in the two groups studied showed the mean HDL (1.12 ± 0.65mmol/L; 1.45 ± 0.40mmol/L) and LDL (2.47 ± 1.09mmol/L; 3.01 ± 1.05mmol/L) in HAART naïve and HAART experienced group respectively (Table 2).

**Table 2:** comparative analysis of the biochemical, viral and atherogenic indices among the studied participants

Variables	HAART naïve group (mean ± SD n=78)	HAART experienced group (mean ± SD n=118)	P value
**Lipid profile**			
Total cholesterol (mmol/l)	4.73 ± 1.44	4.79 ± 0.99	0.741
Triglyceride (mmol/l)	1.38 ± 0.72	1.58 ± 0.97	0.122
High density lipoprotein-c (mmol/l)	1.12 ± 0.65	1.45 ± 0.40	<0.001*
Low density lipoprotein-c (mmol/l)	2.47 ± 1.09	3.01 ± 1.05	<0.001*
**Biochemical and viral parameters**			
Median (IQR) glucose (mmol/l)	5.02 (4.65 - 6.00)	5.02 (4.58 - 5.57)	0.867
Median (IQR) CD4 (cells/μL)	240.00 (107.00 - 431.00)	589.00 (447.00 - 736.00)	<0.001*
Median (IQR) HIV V/L (copies/ml)	6724.00 (200.00 - 50615.00)	50.50 (28.00 - 230.00)	<0.001*
Mean non HDLc (mmol/l)	3.61 ± 1.40	3.34 ± 0.98	0.185
Median (IQR) TG/HDLc	1.00 (1.00 - 2.00)	0.92 (0.62 - 1.40)	0.200
**Renal indices**			
Mean creatinine (μmol/l)	75.36 ± 27.52	85.46 ± 25.08	0.009*
Mean eGFR (ml/min/1.73m2)	112.67 ± 49.35	96.91 ± 26.11	0.004*
**Athrogenic indices**			
Median (IQR) cardiac risk ratio (CRR)	4.50 (3.00 - 6.00)	3.30 (2.65 - 4.08)	<0.001*
Mean CRI - II	2.74 ± 1.78	2.90 ± 7.59	0.859
Median (IQR) AC	3.50 (2.25 - 5.00)	2.29 (1.64 - 3.06)	<0.001*
Median (IQR) QRISK score	0.00 (0.00 - 1.00)	1.50 (0.60 - 3.10)	<0.001*

P-value ≤0.05 is considered statistically significant; non-parametric values as median (IQR)

**Assessment of CD4, viral load and CRR and QRISK3 score among the groups studied:** the median CD4 count (IQR) in HAART naïve group and in HAART experienced group were 240.00 (107.00 - 431.00) cell/μL; and 589.00 (447.00 - 736.00) cell/μL respectively. The median viral load (IQR) was 6724.00 (200.00-50615.00) (copies/mL); and 50.50 (28.00-230.00) copies/mL for HAART naive and HAART experienced group respectively. The median (IQR) cardiac risk ratio (CRR) were 4.50 (3.00 - 6.00) and 3.30 (2.65 - 4.08) for HAART naïve and HAART experienced groups respectively. The risk of CVD events based on QRISK3 varied with a median 1.50 (0.60 - 3.10) among HAART experienced group.

**Assessment of renal function in groups studied:** mean serum creatinine (μmol/l) in HAART experienced and HAART naive participants were: 85.46 ± 25.08 and 75.36 ± 27.52 respectively, while the mean eGFR in HAART experienced and in HAART naïve were (96.91 ± 26.11 ml/min/1•73m^2^) and (112.67 ± 49.35 ml/min/1.73m^2^) respectively. Using Pearson´s and Spearman rank correlation coefficient (Table 3), plasma triglyceride level showed a positive association with CRI-I (r=0.426) whereas HDLc and CD4 exhibited negative associations with CRI-I (r=-0.599 and -0.171) respectively. LDLc, BMI and duration of HAART treatment all exhibited positive associations with the CD4 (r=0.170, r=0.345 and r=0.298) respectively. There was a negative association between CD4 and HIV-viral load.

**Table 3:** correlation between HIV-1 viral load, CD4 count and CRR in HAART experienced volunteers

Variables	r-value	p-value
TG (mmol/l) and CRI-I (CRR)	0.426	<0.001*
HDLc (mmol/l) and CRI-I	-0.599	<0.001*
CD4 count and CRI-I	-0.171	0.031*
LDLc (mmol/l) and CD4 count	0.170	0.017*
BMI (kg/m2) and CD4 count	0.345	<0.001*
Duration of HAART usage and CD4 count	0.298	<0.001*
CD4 count and HIV - 1 viral load	-0.263	<0.001*

* p<0.05 HAART: highly active anti-retroviral therapy; HIV: human immunodeficiency virus; CD: cluster of differentiation; CRR: cardiac risk ratio

The proportion of cardiovascular disease risk factors in the HAART experienced and HAART naïve groups (Table 4) show that increased LDLc was observed in 44 of the participants out of which 33 (28.0%) were HAART experienced while 11 (14.1%) were HAART naïve. Hypertension was a major risk factor in 40 (20.4%) volunteers that participated in this study, out of which 34 were found among HAART experienced (representing 17.35% of the participants and 28.8% of HAART experienced subgroup) compared to 6 (representing 3.06% of the participants and 7.7% of HAART naïve subgroup) observed among the HAART naive group. Among the indices, AIP had 3.6 times increased (combined medium and high) risk, while QRISK3 shows 6 times as much risk in HAART experienced participants compared to HAART naive participants. The atherogenic/glomerular indices by gender, showed that the prevalence of increased atherogenic coefficient (AC), in participants from the two groups was 71 (36.2%); out of which 36 were males while the remaining 35 were females respectively. The prevalence of abnormal estimated glomerular filtration rate (eGFR) was however found to be 7.6% out of which females accounted for 6.1%.

**Table 4:** proportion of cardiovascular disease (CVD) risk factors among HAART-naïve and HAART-experienced group

Risk factor for CVD	Overall n (%)	Those with risk n (%)	HAART experienced group n (%)	Those with risk n (%)	HAART naïve group n (%)	Those with risk n (%)	p-value
LDLc (mmol/l)	196 (100)	44 (22.4)	118 (100)	33 (28.0)	78 (100)	11 (14.1)	0.016*
Overweight/obesity	196 (100)	113 (57.7)	118 (100)	79 (67.0)	78 (100)	44 (56.4)	0.032*
Hypertension	196 (100)	40 (20.4)	118 (100)	34 (28.8)	78 (100)	6 (7.7)	<0.001*
CRI -1	196 (100)	37 (18.9)	118 (100)	11 (9.3)	78 (100)	26 (33.3)	<0.001*
CRI -11	196 (100)	29 (14.8)	118 (100)	15 (12.7)	78 (100)	14 (17.9)	0.314
AC	196 (100)	71 (36.2)	118 (100)	33(28.0)	78 (100)	38 (48.7)	0.003*
AIP (medium risk)	196 (100)	15 (7.7)	118 (100)	15 (12.7)	78 (100)	0 (0.0)	<0.001*
AIP (high risk)	196 (100)	54 (27.6)	118 (100)	39 (33.1)	78 (100)	15 (19.2)	<0.001*
QRISK3 score	196 (100)	7 (3.6)	118 (100)	6 (5.0)	78 (100)	1 (1.3)	0.355
eGFR (renal disease risk)	196 (100.0)	69 (35.3)	118 (100.0)	45 (38.1)	78 (100.0)	24 (30.7)	0.060
**Risk stratification by gender total participants**			**Male n (%)**		**Female n (%)**		**p-value**
AC	196 (100)		71 (100)		125 (100.0)		
Normal	125 (63.8)		35 (49.3)		90 (72.0)		
High	71 (36.2 M=18.3% F=17.9%)		36 (50.7)		35 (28.0)		<0.001*
eGFR(ml/min/1.73m2)	196 (100)		71 (100)		125 (100.0)		
Normal	181 (92.4)		68 (95.7)		90 (72.0)		
Abnormal	15 (7.6 M=1.5%F=6.1%)		3 (4.3)		35 (28.0)		<0.001*

LDLc: low density lipoprotein cholesterol; CRI-I: Castelli's risk index-I; CRI-II: Castelli's risk index-II; AC: atherogenic coefficient; AIP: atherogenic index of plasma; eGFR: estimated glomerular filtration rate

The proportion of risk factors by age stratification (Table 5) showed that the prevalence of hypertension was 20.4%, with participants older than 45 years (>45 years), having greater than 3 times CVD risk than the participants younger than 45 years of age. Prevalence of hypertriglyceridemia was 31.1% (61) in all the participants. However, participants older than (>) 45 years who exhibited hyperglycemia were 74 representing 16.33% of the hypertriglyceridemia in all the participants and 43.2% of the participants in this age category. Participants below the age of 45 years who presented hypertriglyceridemia were 29. This represented 14.80% of the study participants and 27.9% of the participants in that age group. The predisposition to abnormal eGFR, was 35.2%. Participants >45 years (47.3%) were more likely to be predisposed to a CKD event than those <45 years of age (27.9%). Based on QRISK3 calculation, participants older than 45 years were about 9.5 times more probable to experience a CVD event (medium and high risk) in older age (>45 years) than the younger (<45 years) age.

**Table 5:** statistically significant risk factors by age stratification among participants

Risk factor for CVD	Overall	<45 (years)	>45 (years)	p-value
Hypertension	196 (100.0)	122 (100.0)	74 (100.0)	<0.001*
Normotensive	156 (79.6)	108 (88.5)	48 (64.9)	
Hypertensive	40 (20.4)	14 (11.5)	26 (35.1)	
Triglyceride (mmol/l)	196 (100.0)	122 (100.0)	74 (100.0)	0.004*
Normal	135 (68.9)	93 (76.2)	42 (56.8)	
High	61 (31.1)	29 (23.8)	32 (43.2)	
eGFR (ml/min/1.73m2)	196 (100.0)	122 (100.0)	74 (100.0)	0.005*
Normal	127 (64.8)	88 (72.1)	39 (52.7)	
Abnormal	69 (35.2)	34 (27.9)	35 (47.3)	
QRISK3 score	196 (100.0)	122 (100.0)	74 (100.0)	0.003*
Normal (low risk)	189 (96.4)	122 (100.0)	67 (90.5)	
Abnormal (medium and high risk)	7 (3.6)	0 (0.0)	7 (9.5)	

* p<0.05; triglyceride; eGFR: estimated glomerular filtration rate

## Discussion

Cardiovascular diseases (CVD) are widely acknowledged complication of HIV infection. Most of the traditional risk factors observed in the general population are also observed among HIV infected individuals with an increased incidence of CVD and its main risk factors especially hypertension, diabetes, obesity and dyslipidemia [10]. In this study, the mean age of HAART experienced participants was observed to be higher (p<0.05) than the HAART naïve group (Table 1). This can be attributed to the positive impact of HAART treatment on the health status of the treated group which possibly contributed to improvement in their life expectancy [11]. Because some of the persons on ART might have been on treatment a couple of years back, they are likely to be older. Moreover, many of the individuals in treatment naïve group were just coming to true realization of their HIV sero-positive status, majority of these individuals were younger in age. A current epidemiology of human immunodeficiency virus infections in Nigeria put the prevalence of HIV at 1.4% in person´s age 15-49 years from the recent Nigeria HIV/AIDS indicator and impact survey (NAIIS) [12]. The mean weight and body mass index (BMI) were also higher in HAART experienced group due to improved immune system-a consequence of the positive impacts of the treatment. In addition to this, the mean systolic blood pressure (SBP) and diastolic blood pressure (DBP) were higher in HAART experienced participants when compared with the HAART naïve group. Chow *et al*. [13] in their study reported that untreated HIV sero-positive individuals tend to have lower blood pressure (BP) whereas, upon normalization of immune status and suppression of replication, there is elevation of blood pressure. In this study, 39.8% of the study population were HAART naïve (about to commence treatment) as at the time of their inclusion into this study, 60.2% were on HAART treatment. In addition to this, 36.2% and 63.8% of the participants were males and females respectively (Table 1). This observation is in accord with previous report which indicated that women constituted more than half of all people living with HIV [14]. This may be due to their unequal cultural, social and economic status including, gender inequalities with gender based and intimate partner violence; all these are common in some developing economy like Nigeria where this study was conducted [15].

Moreover, there was no significant difference in the plasma glucose concentration of the HAART experienced and HAART naïve group as at the time of this study (Table 2). Analysis of the lipid profile of the study participants showed that HAART experienced patients had significantly higher HDL-c and LDL-c when compared to HAART naïve group. The mean HDL-c and the LDL-c (the major predictors of athero-protective and athero-genic status) were significantly higher in the HAART experienced group. This observation concurred with the previous study by Manuthu *et al*. [16]. The high prevalence of low HDL-c among HAART naïve individuals may be a consequence of altered lipid metabolism which occurs in HIV infection. Riddler *et al*. [17] suggested that HIV sero-conversion resulted in reductions of TC, LDL-c and HDL-c levels and that initiation of HAART increased both TC and LDLc levels but not HDLc. Low HDLc levels in HAART naive group as at the time of this study may suggest that HIV infection could be associated with modified HDLc metabolism, re-directing cholesterol to the Apo B-containing lipoprotein and likely reduces the functionality of reverse cholesterol transport [18]. However, Negredo *et al*. [19] reported that non-nucleotide reverse transcriptase inhibitors (NNRTI) raises HDLc levels substantially as immunological status improves which may provide a potential long term beneficial effect on cardiovascular risk profile. NNRTI could impact beneficial effects on lipid profile in addition to reducing viral replication and improving immunological status of infected patients.

Furthermore, CD4 values in HAART experienced patients were significantly higher than the HAART naïve group whereas the HIV-1 viral load, cardiac risk ratio (CRI-I) and AC values were higher among HAART naïve individuals. Thus, a significant increase in CD4 lymphocytes among HAART experienced patients suggest the positive impacts of long term HAART usage in the management of HIV patients. It has been suggested that the use of HAART was associated with deliberate attempts to gain weight. However, there were debilitating impacts of HIV on the HAART naïve volunteers leading to HIV induced loss of immunity as a result of extremely high HIV RNA viral load - a proxy for the average transmission risk in a given population [20] as well as decrease in CD4 count, malnutrition and opportunistic infections with loss of weight (Table 1) as one of the significant consequences [21]. The outcome of this study with respect to CD4 count among HAART treated patients agrees with the previous study by Le Moing *et al*. [22], who reported that maintaining viral suppression results in greater increase in CD4 cell counts in the long term. Lower CD4 count among HIV-naïve volunteers was observed in this study (Table 2). A low CD4 count, immunosuppression and higher levels of HIV viraemia have been associated with higher risk of myocardial infarction and stroke [23,24]. Viraemia is thought to mediate dyslipidaemia through promoting lipid peroxidation which is responsible for alteration of cholesterol metabolism [25].

In addition to this, the degree of association between the continuous variables studied was evaluated (Table 3). It was observed that triglyceride was positively associated (p<0.05) with CRI-I; suggesting that an increase in triglycerides enhances increase in cardiac risk ratio (CRR). Increase in CRI-I on the other hand has been recognized as a strong marker of future risk of CVD [5]. A negative association (p<0.05) between CRI-I and HDL-c was also observed. This observation suggests that an improved HDL-c in HAART experienced individuals reduces cardiac risk ratio. Previous piece of literature has shown that reduced CRI-I leads to decrease in risk of CHD [6]. A negative correlation between CD4 count and CRI-I was also observed. It appears that an increase in CD4 count [22] which is a marker of improved immunity as a result of the positive impact of HAART treatment suppresses CRI-I. Also, a positive association was observed between CD4 and LDL-c, BMI, duration of HAART treatment. This observation suggests the positive impact of prolong HAART treatment as it promotes improved CD4 count which invariably improves the body mass index as well as increasing the risk of higher LDL-c.

Conversely, risk of hypertension, increased LDLc and risk of cardiovascular disease (CVD) (as predicted by QRISK score) were 5.6 times, 3 times and 6 times higher respectively among HAART treated patients (Table 4), suggesting a predisposition to HAART induced atherogenicity. Atherogenic coefficient (AC) was observed to be more prevalent in the male gender (Table 4) with 50.7% having high atherogenicity predictor against female participants 28%. This observation agrees with the previous study by Kappert *et al*. [26]. This observation was however, at variance with the study by Towfighi *et al*. [27]. Similarly, risk of chronic renal disease was higher among the HAART experienced participants as shown by a lower estimated glomerular filtration rate (Table 2). This observation concurs with the previous study by Schwartz *et al*. [28] and may be due to nephrotoxic consequences of several ARTs as described by Mocroft *et al*. [29]. The risk of renal failure was also higher in female HAART experienced patients when compared with males (6.1% vs 1.5% among total number of participants) as shown in Table 4. This finding is also in accordance with the previous study by Mekuria *et al*. [30].

The prevalence of hypertension (28.8%) in HAART experienced group (Table 4) when compared with the HAART naïve group (7.7%) corroborates with the previous study by Mashinya *et al*. [31]. The outcome of this study with regards to the prevalence of hypertension among HAART experienced was higher than a similar ART cohort of younger adults study by Sander *et al*. [32]. The difference may be attributed to older ages of the participants in this study, HAART treatment, as well as the duration of HAART treatment, lower viral loads and genetic variations among the population studied. Furthermore, the HAART experienced group had significant increase in AIP risk level (Table 4). Bhardwaj *et al*. [33] reported elevations in AIP was a probable predictor of CVD. However, Nwagha *et al*. [8], opined that in a situation where normal triglyceride (an atherogenic risk) is observed, AIP may possibly serve as the diagnostic alternative, whereas da Luz *et al*. [34] established that a high CRI-1 (CRR) strongly predicts coronary disease.

QRISK3 for the prediction of CVD, categorized as low risk was 5.0% among HAART experienced group (Table 4). This was however lower than the finding by Kazooba *et al*. [35], that reported a prevalence of 16.6%. The possible reason for this variation may be due to different risk algorithm used in these studies as in their study, the prediction of future risk of CVD was calculated using Framingham risk score (FRS). The use of AIP as an index of dyslipidaemia among HAART experienced individuals showed 12.7% moderate risk while 33.1% were at high risk of atherosclerosis and CHD despite their normal mean level of TC and LDLc. In similar studies, Noumegne *et al*. [36] reported 32.5% while, Wekesa *et al*. [37] reported 25.0% risk. These findings support the assertion that HIV and ART were risk factors of metabolic complications and thus likely to predispose patients to an increased risk of dyslipidaemia and its correlates. Furthermore, the risk stratification by gender (Table 4) shows that atherogenic coefficient (AC) was higher in male whereas the risk of chronic kidney failure was significantly higher in female participants.

Conversely, risk factors by age stratification of HIV infected individuals were examined (Table 5). It was observed that as HAART improves survival (life expectancy) of the patients treated, there was a potential for increased incidence of CVD as observed by significant risk of hypertension and QRISK3 score in participants older than 45 years and CKD (as measured by significant decrease in eGFR) in a population aging with HAART. HIV-sero-positive people not uncommonly have high predicted CVD and/or CKD risk; these interact to create substantial risks for future morbid events [38]. Ryom *et al*. [39] reported that the effects of comorbidity appeared greater than the effects of the sum of risk of each disease; leading to more severe illness, poorer prognosis, and premature death. Thus, this underscores the need for CVD and CKD risk factors assessment before initiation of HAART followed by periodic monitoring while on the treatment. Doing this will ensure prompt detection and management of predisposing risk factors and the prevention of subtle but sudden cardiovascular and/or renal events, particularly in people surviving and aging with HIV infection.

## Conclusion

Dyslipidaemia, atherogenic disorders, renal failure and increase risk of cardiovascular diseases appeared to be common among people living with human immunodeficiency virus. This is however more pronounced among HAART experienced patients; possibly due to increase in life expectancy of this group of individuals. With increasing years of survival of HAART experienced patients, predisposition to increased atherogenic disease conditions such as low HDL-c, hyper-triglyceridaemia, increased LDL-c, hypertension and obesity seem to be on the increase. From this study, it appeared that atherogenicity was more prevalent in the male gender. Glomerular diseases appeared more in the female gender. HAART experienced participants had greater risk of CVD as observed from this study. Thus, there is need for a thorough monitoring of patients on HAART treatment.

**Limitation of the study:** this study was limited to individuals who have been infected by human immunodeficiency virus as the outcome from this study may not be the same with individuals who are not infected with the virus. Also, there is a possibility of underestimation of cardiovascular diseases with the risk assessment studied. Effect of diet which is also a factor was not considered in this study.

### What is known about this topic

- CVD and its increasing risk factors are a leading cause of morbidity and mortality globally;
- HIV virus on its own and its metabolism predisposes HIV infected patients to develop CVD risk factors;
- HAART treatment prevents viral replication thereby restoring the health of the infected individuals.

### What this study adds

- Risk of CVD, particularly, dyslipidaemia as well as, renal failure which is more predominant in the female gender are common in HAART treated patients in our environment;
- Atherogenic indices are invaluable instruments in evaluating CVDs among HIV patients on HAART management;
- QRISK score for the prediction of cardiovascular diseases is clearly useful in predicting future predisposition to CVDs in HIV infection and in HAART treatment.
